# Natural Occurrence of Nivalenol, Deoxynivalenol, and Deoxynivalenol-3-Glucoside in Polish Winter Wheat

**DOI:** 10.3390/toxins10020081

**Published:** 2018-02-13

**Authors:** Marcin Bryła, Edyta Ksieniewicz-Woźniak, Agnieszka Waśkiewicz, Krystyna Szymczyk, Renata Jędrzejczak

**Affiliations:** 1 Department of Food Analysis, Prof. Waclaw Dabrowski Institute of Agricultural and Food Biotechnology, Rakowiecka 36, 02-532 Warsaw, Poland; edyta.wozniak@ibprs.pl (E.K.-W.); krystyna.szymczyk@ibprs.pl (K.S.); renata.jedrzejczak@ibprs.pl (R.J.); 2 Department of Chemistry, Poznan University of Life Sciences, Wojska Polskiego 75, 60-625 Poznan, Poland; agat@up.poznan.pl

**Keywords:** deoxynivalenol, deoxynivalenol-3-glucoside, masked mycotoxins, nivalenol, winter wheat

## Abstract

The presence of mycotoxins in cereal grain is a very important food safety factor. The occurrence of “masked” mycotoxins has been intensively investigated in recent years. In this study, the occurrence of nivalenol, deoxynivalenol-3-glucoside, and deoxynivalenol in 92 samples of winter wheat from Polish cultivars was determined. The frequency of the occurrence of deoxynivalenol and nivalenol in the samples was 83% and 70%, respectively. The average content of the analytes was: for deoxynivalenol 140.2 µg/kg (10.5–1265.4 µg/kg), for nivalenol 35.0 µg/kg (5.1–372.5 µg/kg). Deoxynivalenol-3-glucoside, the formation of which is connected with the biotransformation pathway in plants, was present in 27% of tested wheat samples; its average content was 41.9 µg/kg (15.8–137.5 µg/kg). The relative content of deoxynivalenol-3-glucoside (DON-3G) compared to deoxynivalenol (DON) in positive samples was 4–37%. Despite the high frequency of occurrence of these mycotoxins, the quality of wheat from the 2016 season was good. The maximum content of DON, as defined in EU regulations (1250 µg/kg), was exceeded in only one sample. Nevertheless, the presence of a glycosidic derivative of deoxynivalenol can increase the risk to food safety, as it can be hydrolyzed by intestinal microflora.

## 1. Introduction

Wheat is a basic ingredient used for food preparation around the world. Poland, with approximately 11 million metric tons cultivated each year, is the fourth largest producer of this crop in the European Union (after France, Germany, and the United Kingdom) and 15th in the world [1]. Cereal plants are vulnerable to infections of pathogenic fungi of the *Fusarium* genus. *Fusarium* head blight (FHB) is an infection widespread in wheat-producing countries; it is induced mainly by *F. graminearum*, however *F. culmorum* and *F. avenaceum* can also be dominating species. The optimal conditions for the fungal infection and propagation of these fungi are moderate temperature and high air humidity [2]. The geographic occurrence of *F. graminearum* and *F. culmorum* may vary depending on the climatic conditions (temperature and relative air humidity): *F. graminearum* occurs mostly in warmer and more humid regions (e.g., North America, eastern Europe, Australia, southern China), while *F. culmorum* occurs mainly in colder climatic regions (e.g., Western Europe) [3]. This is not a rule, thus in some regions, sometimes the main FHB-inducing factors were *F. culmorum*, *F. avenaceum*, and *F. poae* [4]. FHB leads to economic losses caused by the decrease in crop yields, but also can have an impact on food safety. This is connected with accumulation of mycotoxins in grains. Nivalenol (NIV) and deoxynivalenol (DON), classified as type B trichotecenes, are fungal metabolites present in agricultural products [5,6].

NIV is not found in food as commonly as DON; however, it demonstrates higher toxicity in animal studies. The LD50 values for DON and NIV in tests in mice were 78 and 39 mg/kg, respectively [7]. The toxicity of NIV is often compared to the toxicity of DON; however, the amount of toxicological data on NIV impact is much lower. On the molecular level DON and NIV, similarly to other trichotecenes, show many limiting effects on the primary eukaryotic cell metabolism including inhibition of protein, DNA, and RNA synthesis [8,9].

**Figure 1:**
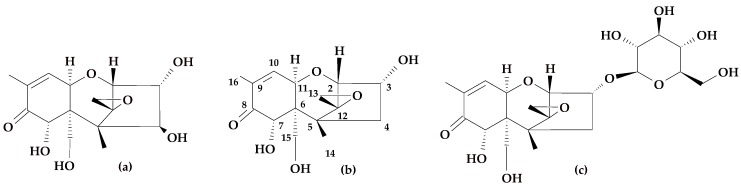
Structural formulas: (**a**) nivalenol (NIV); (**b**) deoxynivalenol (DON); and (**c**) deoxynivalenol-3-glucoside (DON-3G).

DON acts as a virulence factor in the development of FHB and facilitates the spread of fungus from the infection site [10]. After synthesis of this mycotoxin in the infected tissue, DON inhibits protein synthesis, disrupts signal transmission, and eventually causes cell death [11]. Plants possess developed detoxication systems. In the case of cereal plants in the scope of these reactions, there is a group of mycotoxins, which undergo biological modification. The modification is made by two major reactions. In the first stage xenobiotics are oxidized or hydrolyzed; the second stage is a conjugation in which the xenobiotics functional group binds with glucose, malonic acid, or glutathione. In the reaction of the second phase of detoxication deoxynivalenol-3-glucoside (DON-3G), which is the main DON metabolite, may be formed [12,13]. The structures of these compounds are presented in Figure 1. Mycotoxin metabolites that were synthesized in this manner are described as “masked mycotoxins” [14,15]. DON-3G was detected in naturally contaminated wheat and maize for the first time in 2005 [16]. Afterwards, the presence of the DON-3G biosynthesis pathway was confirmed also in durum wheat and barley [17,18]. DON-3G, compared to the basic analogue, exhibits lower synthesis of ribosomal protein of wheat in in vitro conditions [19]. In the biosynthesis of this metabolite, uridine-5'-diphospho-glucuronosyltransferase (UDP-glucosyltransferase) participates, which catalyses the transfer of glucose from UDP-glucose to the hydroxyl group of DON on the third carbon atom. The DON-resistant wheat cultivars are more efficient in conversion of DON to DON-3G than more susceptible cultivars. Thus, it is believed that implementing a detoxication mechanism based on glicosylation in cultivars susceptible to *Fusarium* will increase wheat resistance to FHB [20]. One of the most recent attempts to increase the resistance was using transgenic wheat containing barley UDP-glucosyltransferase gene (HVUGT13248), which demonstrated a high level of resistance [21]. The majority of the studies on the toxicological features of DON-3G were conducted in vitro. It is still not clear how it impacts living organisms. In general, it is stated that DON-3G is less toxic than its parent toxin. It is resistant to digestion processes in digestive systems and is not absorbed by the intestinal epithelium, although it can by hydrolyzed to DON or deepoxy-deoxynivalenol (DOM-1) by intestinal microflora [22,23,24].

Because of the reasons listed above, knowledge about the natural occurrence of masked mycotoxins in wheat samples is very important from the viewpoint of food safety. In Poland, there are no routine analyses of DON-3G (in mills or elevators). Similarly, due to the lack of legal regulations, there is no obligatory NIV screening. The aim of this study was to evaluate natural occurrence of NIV, DON and DON-3G (a product of DON metabolism in plants) in wheat grain harvested in Poland.

## 2. Results

### 2.1. Sample Processing

Wheat samples fortified with analytical standards added to determine method recovery and precision were processed identically to real samples. 2 g of ground wheat grain and 8 mL of de-ionised water put into a 50 mL falcon tube were first homogenized (Unidrive X 1000 homogenizer manufactured by CAT Scientific, Inc., Pase Robles, CA, USA) for 2 min, then centrifuged (MPV, Med. Instruments, Warsaw, Poland) at 10,730 × *g* for 10 min. 3 mL of the extract dissolved with 3 mL of phosphate buffered saline (PBS) was again centrifuged at 10,730 × *g* for 10 min. 5 mL of the extract was passed through a DON-NIV (wide-bore, WB) immunoaffinity column (Vicam, Watertown, MA, USA) at a speed of 1–2 drops/s. Next, the column was rinsed with 10 mL of PBS and 10 mL of de-ionised water at a speed of 2–3 drops/s. Analytes washed out of the column with first 0.5 mL of methanol then with 1.5 mL of acetonitrile were collected into a reaction vial. The solvent was evaporated in a stream of nitrogen, and the residues were re-dissolved in 300 µL of 10% acetonitrile solution.

**Figure 2:**
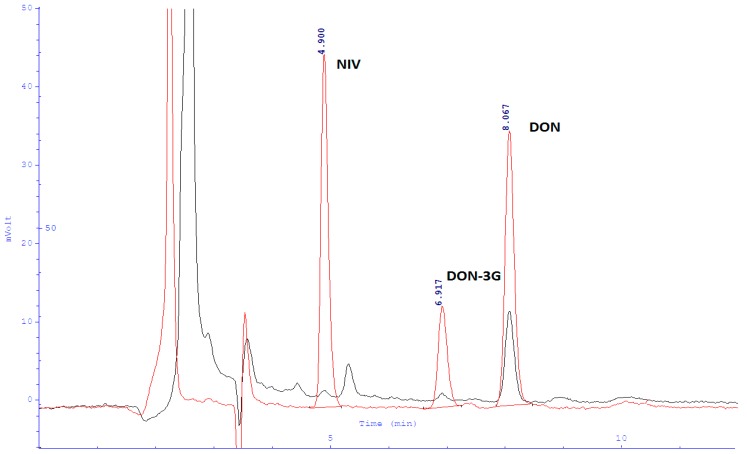
Black: chromatogram taken from a naturally contaminated wheat sample. Red: chromatogram of 480 µg/kg NIV, DON, and DON-3G standards.

The samples ready for injection were filtered through a nylon syringe filter with 0.45 µm pore diameter. Each sample was analysed twice; two independent repetitions were sufficient since the used immunoassay columns (IAC) columns (Vicam, Watertown, MA, USA) feature very high sample purification degree and repeatability. Chromatograms taken from naturally contaminated wheat samples are shown in Figure 2, respectively, on top of chromatograms of mixtures of NIV, DON and DON-3G standards.

### 2.2. Validation of the Analytical Method

10 mixtures made by successive dilution of NIV/DON/DON-3G standard solutions were prepared and analysed to construct calibration curves. The dilution ranges were 0.017–2.018/0.017–2.010/0.017–2.016 µg/mL, respectively. Linearity ranges were 4.8–968.6/10.1–964.8/15.0–967.7 µg/kg, respectively. The obtained R^2^ coefficients of the curves were high (0.9937/0.9965/0.9988, respectively). The limits of quantification (LOQ) (concentrations, at which the S:N ratio dropped to 10) were 16.8/33.1/50 µg/kg, respectively. The limit of dection (LOD) thresholds (concentrations, at which the S:N ratio dropped to 3) were 4.8/10.1/15.0 µg/kg, respectively.

Recovery (*R*) and repeatability (precision) (relative standard deviation, RSD) of the method was checked for three fortification levels: 225/450/900 µg/kg. Ground blank wheat grain was used in the validation experiment. Depending on the level, the analyte, the obtained *R* values ranged from 76.9 to 109.4% (see Table 1), while the RSD values ranged from 2.2 to 7.9%. The ranges are quite satisfactory.

### 2.3. Occurrence of Mycotoxins in Wheat Grain

**Figure 3:**
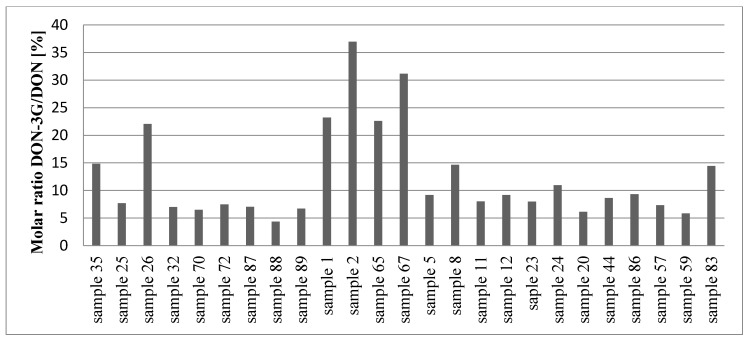
The relative content of DON-3G compared to DON (%) in positive samples.

The presence of NIV/DON in wheat grain proves the plants must have been infected with the *Fusarium* fungi. DON-3G is a product of plant metabolism produced from DON by plant enzymes. NIV (above the method LOD threshold) was found in 70% of the studied wheat samples, DON—in 83% of the samples (Table 2). DON levels (mean 140.2, range 10.5–1265.4 µg/kg) were clearly higher than NIV levels (mean 35.0, range 5.1–372.5 µg/kg). The maximum acceptable level of DON in wheat specified in the WE 1881/2006 regulation (1250 µg/kg) was exceeded in one sample. DON-3G was found in only 27% of the studied wheat samples, a surprisingly low fraction compared to the fraction of samples contaminated with DON. Also, DON-3G levels were clearly lower than DON levels: mean 41.9 µg/kg, range 15.8–137.5 µg/kg. The DON-3G/DON ratios are shown in the bottom row of Figure 3; their range is 4–37%.

**Figure 4:**
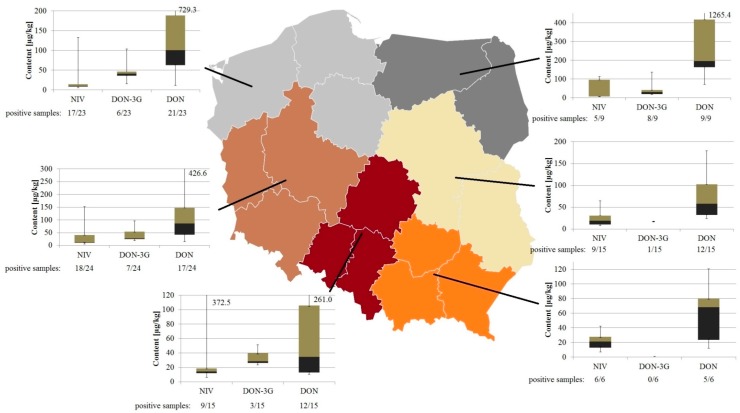
NIV, DON and DON-3G concentrations (min, lower quartile, median, upper quartile, max) found in wheat sampled in six regions of Poland. Number of positive samples/total number of samples fractions are shown under bar charts.

NIV, DON and DON-3G concentrations (min, lower quartile, median, upper quartile, max) found in wheat sampled in six regions of Poland are charted in Figure 4. The ratios of the number of positive samples to total number of samples collected in the given region are shown under each bar chart.

Generally speaking, winter wheat grown in Poland during the 2016 growing season was contaminated with NIV and DON (including DON-3G, metabolite of the latter) at quite low levels. The highest contamination levels were found in 9 samples from north-east Poland, minimum–median–maximum concentrations were: 6.0–7.4–112.4/71.3–196.4–1265.4/16.4–30.7–137.5 µg/kg for NIV/DON/DON-3G, respectively. The lowest pollution was found in 6 samples from south-east Poland, minimum-median-maximum concentrations were: 6.8–21.0–42.2/12.2–68.1–120.3 µg/kg for NIV/DON, respectively. DON-3G was not found in any of the samples from the south-east. 

## 3. Discussion

Capability to improve method selectivity by removing interfering compounds is one of big advantages of immunoassay columns (IAC). Since samples get concentrated, LOD/LOQ thresholds for analytes may be improved. Highly purified samples produced by IAC columns used to isolate the to-be-determined analytes from extracts may be readily analysed with the help of the liquid chromatography (LC) technique using various detectors. In our analyses it was an Ultraviolet–Visible (UV-VIS) detector (Knauer, Wissenschaftliche Geräte GmbH, Berlin, Germany) operated at 218 nm wavelength. The DON-NIV WB columns (Vicam, Watertown, MA, USA) applied by us contain some antibodies allowing them to feature a high cross-reactivity for modified mycotoxins. Therefore they enabled simultaneous analyses of NIV, DON, and DON-3G. Advantages of the columns have recently been successfully utilized also by other authors (Trombete et al. [25], Geng et al. [26], Yoshinari et al. [27]).

Mycotoxins produced by *Fusarium* fungi are commonly found in grain cultivated on every continent. A high fraction of our samples were NIV- and DON-positive (70% and 83% of the samples, respectively), while DON-3G was found in only 27% of the samples. Concentration means (ranges) were quite low: 35.0 (5.1–372.5)/140.2 (10.5–1265.4)/41.9 (15.8–137.5) µg/kg respectively for NIV/DON/DON-3G. While literature data on the fraction of positive samples are similar, concentrations found by other authors were somewhat higher. It may be concluded that wheat grain harvested in 2016 in Poland was polluted with the above mycotoxins to a quite low degree. The extent of contamination depends on climatic conditions prevailing in any given growing season at any given geographic location of the cultivation site. The content of DON-3G, natural product of metabolism of DON under the influence of plant enzymes, directly depends on the wheat genotype. The role of the glycosylation mechanism coded in genotypes of wheat and other corns in plant resistance to FHB is worthy of more detailed studies [28]. Dong et al. studied conversion of DON into DON-3G in various wheat varieties cultivated in the Jiangsu region of China during 2015 and 2016 growing seasons [29]. Wheat cultivated in the lower course of the Yangtze-Huai rivers has always been vulnerable to FHB. 100% of the studied samples were DON-polluted at mean concentrations 2087/2061 µg/kg for 2015/2016, respectively. Respective figures for DON-3G were: 96%/97% positive samples, 545/819 µg/kg mean concentration for 2015/2016, respectively. The DON-3G/DON ratio ranged from 5 to 84% in 2015 samples (mean 30%) and from 0 to 71% in 2016 samples (mean 31%). Concentrations and relative proportions of individual analytes depended on plant genotype and cultivation site. Palacios et al. studied 84 samples of durum wheat cultivated in Argentina [30]. DON was found in 100% of the samples at concentrations ranging from 50 to 9480 µg/kg. DON-3G was found in 94% of the samples at concentrations ranging from 50 to 850 µg/kg. DON-3G/DON ratios ranged from 6 to 22%. Bryła et al. studied 45 samples of wheat cultivated in three regions of Poland during the 2013/2014 growing season [31]. DON was found in 100% of the samples at concentrations ranging from 82 to 2975 µg/kg. DON-3G was found in 44–78% of samples (depending on the cultivation region) at concentrations ranging from 40 to 356 µg/kg. Trombete et al. studied 17 samples of wheat cultivated in Portugal [25]. NIV was found in 29.5% of the samples at concentrations ranging from 31.3 to 140.6 µg/kg. DON was found in 58.8% of the samples at concentrations ranging from 31.3 to 325.8 µg/kg. DON-3G was found in 41.2% of the samples at concentrations not exceeding 41.7 µg/kg. Calori-Domingues et al. studied 745 wheat samples cultivated in Brazil during the 2009 and 2010 growing season [32]. NIV was found in 50% of the samples, mean concentration was below 100 µg/kg. DON was found in 86% of the samples, mean concentration was 1046 µg/kg. De Boevre et al. reported maize polluted with 1100 µg/kg of DON-3G [33]. Similar contamination was reported by Berthiller et al. in wheat polluted with 1070 µg/kg of DON-3G [34]. Dall’Asta et al. studied 150 samples of durum wheat cultivated in northern and central regions of Italy [28]. DON was found in 100% of samples at concentrations ranging from 47 to 3715 µg/kg. DON-3G was found in 83% of samples at concentrations ranging from 46 to 842 µg/kg. DON-3G/DON ratios did not exceed 30%. Rasmussen et al. studied 6 samples of winter wheat, 6 samples of triticale, and 11 samples of oats cultivated in Denmark [35]. NIV was found only in 2 oats samples at concentrations 79 and 98 µg/kg. DON was found in all 6 wheat samples (46–2638 µg/kg), in 5 triticale samples (43–737 µg/kg), and in 9 oat samples (62–2216 µg/kg). DON-3G was found in 3 wheat samples (96, 134 and 342 µg/kg), in one triticale sample (109 µg/kg), and in 5 oat samples (162–287 µg/kg).

## 4. Conclusions

Occurrence of NIV, DON, and DON-3G in 92 samples of various winter wheat cultivars cultivated in the 2015/2016 vegetation season in various regions of Poland was evaluated using the LC chromatography technique with an UV detector. Cross reactivity of antibodies contained within DON-NIV WB immunoassay columns made possible to determine DON-3G (i.e., DON masked form) simultaneously with NIV and DON. IAC effectiveness was checked in a validation experiment.

NIV/DON/DON-3G was identified above the method LOD in 70%/83%/27% of the samples, respectively, but at safely low levels. Mean concentrations of the analytes were (all data in µg/kg): 35.0 (range 5.1–372.5)/140.2 (range 10.5–1265.4)/41.9 (range 15.8–137.5), respectively. The DON-3G/DON molar ratio ranged from 4.3 to 37.0%.

The results obtained in this work are quite important for food safety, especially since literature data on the pollution of food with DON-3G are quite scarce. Although the frequency of occurrence of the discussed mycotoxins was high, the quality of the wheat was generally high. In must be emphasized that DON-3G can increase the risk to food safety, because, as was reported in the literature, it can be hydrolysed in the intestine by microflora.

## 5. Materials and Methods

### 5.1. Reagents/Standards/Columns

Certified reference standards of NIV (100 µg/mL in acetonitrile), DON (100 µg/mL in acetonitrile) and DON-3G (50 µg/mL, in 1:1 acetonitrile: water mixture) were purchased from Romer Labs (Tulln, Austria). Acetonitrile and methanol (high performance liquid chromatography (HPLC) grade) were purchased from Honeywell (Seelze, Germany). Liquid chromatography–mass spectrometry (LC-MS) grade water was purchased from Sigma-Aldrich (St. Louis, MO, USA). DON-NIV immunoaffinity columns WB and phosphate buffered saline (PBS) were purchased from Vicam (Watertown, MA, USA).

### 5.2. Samples

**Figure 5:**
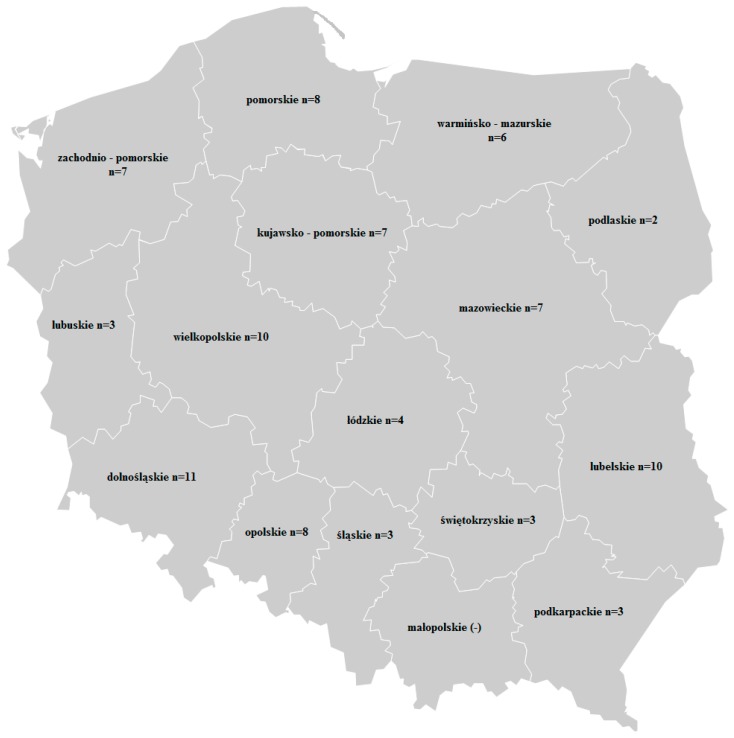
The localization of the origin of the samples.

The 92 samples of winter wheat were collected in 2016 from crops harvested in various regions of Poland. The samples were gathered at collection points of The National Center for Agricultural Support located in 15 voivodeships; the map of them is presented in Figure 5.

### 5.3. HPLC-UV

A Knauer K 1001 HPLC instrument (Knauer, Wissenschaftliche Geräte GmbH, Berlin, Germany) equipped with an autosampler, a thermostat, and a 45 µL injection loop was used. Analytes were separated using a Cosmosil 5C18-AR-II, 4.6 × 250 mm chromatographic column (NacalaiTesque, Kyoto, Japan) kept at 45 °C constant temperature. Separation was done in isocratic mode. The mobile phase was 90:10 *v/v* water: acetonitrile mixture flowing at 1 mL/min. An UV detector manufactured by Knauer was operated at 218 nm wavelength. Knauer Eurochrom HPLC Software ver. 1.65 was used to integrate chromatographic peaks.

## Figures and Tables

**Table 1:** Method recovery *R* (%) and precision RSD (%) for fortified wheat samples.

Compound	Fortification Level (*n* = 3)					
	225 µg/kg		450 µg/kg		900 µg/kg	
	*R*%	RSD%	*R*%	RSD%	*R*%	RSD%
NIV	109.4	2.2	102.3	7.4	78.6	4.0
DON	91.8	2.0	95.0	5.0	76.9	3.4
DON3G	82.8	6.4	81.7	7.9	98.2	5.4

**Table 2:** Concentrations (µg/kg) of NIV, DON, and DON-3G in *n* = 92 studied wheat samples.

Voivodeship		NIV	DON-3G	DON	Voivodeship		NIV	DON-3G	DON
Zachodnio-Pomorskie *n* = 7	positive samples	6 (86%)	1 (14%)	5 (71%)	Dolnośląskie *n* = 11	positive samples	8 (72%)	3 (27%)	10 (91%)
	mean ^1^	37.8	43.3	86.5		mean ^1^	31.1	35.5	137.0
	median ^1^	9.4	43.3	76.3		median	12.0	25.6	88.7
	Min-Max ^1^	5.2–133.5	- ^2^	19.5–188.6		Min-Max	5.1–152.8	24.1–56.9	21.6–426.6
Pomorskie *n* = 7	positive samples	6 (71%)	2 (29%)	7 (100%)	Opolskie *n* = 8	positive samples	5 (63%)	2 (25%)	6 (75%)
	mean ^1^	21.1	42.9	134.1		mean ^1^	112.4	26.0	106.0
	median	14.3	42.9	137.6		median	18.8	26.0	45.7
	Min-Max	10.2–52.0	39.0–46.9	11.7–359.2		Min-Max	14.3–372.5	23.6–28.4	12.7–261.0
Warmińsko-Mazurskie *n* = 6	positive samples	3 (50%)	5 (83%)	6 (100%)	Łódzkie *n* = 4	positive samples	2 (50%)	- ^2^	4 (100%)
	mean ^1^	71.8	50.8	432.0		mean ^1^	9.2	- ^2^	24.5
	median	95.9	28.0	290.2		median	9.2	- ^2^	12.6
	Min-Max	7.0–112.4	16.4–137.5	71.3–1265.4		Min-Max	6.3–12.1	- ^2^	10.5–62.4
Lubuskie *n* = 3	positive samples	3 (100%)	2 (67%)	2 (67%)	Śląskie *n* = 3	positive samples	2 (67%)	1 (33%)	2 (67%)
	mean ^1^	21.1	22.8	49.7		mean ^1^	10.6	51.3	136.2
	median	11.0	22.8	49.7		median	10.6	51.3	136.2
	Min-Max	10.7–41.7	18.8–26.9	47.0–52.3		Min-Max	7.8–13.4	- ^2^	42.2–230.1
Wielkopolskie *n* = 10	positive samples	7 (70%)	2 (20%)	5 (50%)	Świętokrzyskie *n* = 3	positive samples	3 (100%)	- ^2^	3 (100%)
	mean ^1^	48.1	73.1	97.9		mean ^1^	18.3	- ^2^	74.6
	median	17.7	73.1	100.0		median	14.7	- ^2^	79.9
	Min-Max	9.7–150.8	50.3–95.9	15.0–198.8		Min-Max	12.4–27.7	- ^2^	23.7–120.3
Kujawsko-Pomorskie *n* = 9	positive samples	6 (67%)	3 (33%)	9 (100%)	Lubelskie *n* = 10	positive samples	4 (40%)	- ^2^	9 (90%)
	mean ^1^	9.7	51.5	206.6		mean ^1^	37.8	- ^2^	75.0
	median	9.3	35.1	148.9		median	38.2	- ^2^	41.4
	Min-Max	5.2–14.9	15.8–103.6	45.4–729.3		Min-Max	10.0–64.8	- ^2^	27.6–179.5
Mazowieckie *n* = 5	positive samples	5 (100%)	1 (20%)	3 (60%)	Podkarpackie *n* = 3	positive samples	3 (100%)	- ^2^	2 (67%)
	mean ^1^	17.8	17.0	72.5		mean ^1^	25.4	- ^2^	40.2
	median	18.6	17.0	73.8		median	27.2	- ^2^	40.2
	Min-Max	7.8–30.8	- ^2^	23.8–119.9		Min-Max	6.8–42.2	- ^2^	12.2–68.1
Podlaskie *n* = 3	positive samples	2 (50%)	3 (100%)	3 (100%)	TOTAL *n* = 92	positive samples	64 (70%)	25 (27%)	76 (83%)
	mean ^1^	6.7	30.1	223.1		mean ^1^	35.0	41.9	140.2
	median	6.7	33.3	196.4		median	13.6	33.3	83.2
	Min-Max	10.2–52.0	39.0–46.9	11.7–359.2		Min-Max	5.1–372.5	15.8–137.5	10.5–1265.4

^1^ Results expressed in µg/kg; ^2^ Not detected (<LOD).

## References

[1] CSO. Central Statistical Office (2017). Concise Statistical Yearbook of Poland. https://danepubliczne.gov.pl/dataset/5c9f136c-025d-4b82-b03c-d8d7148dfe09/resource/cd90dfe3-1665-4a4d-b034-25e0ead1b389/download/malyrocznikstatystycznypolski2017.pdf.

[2] Duveiller E., Singh P.K., Mezzalama M., Singh R.P., Dababat A.A. (2012). Wheat Diseases and Pests: A Guide for Field Identification.

[3] Simsek S., Burgess K., Whitney K.L., Gu Y., Qian S.Y. (2012). Analysis of Deoxynivalenol and Deoxynivalenol-3-glucoside in wheat. Food Control.

[4] Lenc L., Czecholiński G., Wyczling D., Turów T., Kaźmierczak A. (2015). Fusarium head blight (FHB) and *Fusarium* spp. on grain of spring wheat cultivars grown in Poland. J. Plant Protect. Res..

[5] Tanaka K., Kobayashi H., Nagata T., Manabe M. (2004). Natural occurrence of trichothecenes on lodged and water-damaged domestic rice in Japan. Food Hyg. Saf. Sci..

[6] Schollenberger M., Müller H.M., Rüfle M., Suchy S., Planck S., Drochner W. (2005). Survey of Fusarium toxins in foodstuffs of plant origin marketed in Germany. Int. J. Food Microbiol..

[7] International Agency for Research on Cancer (IARC) (1993). Some Naturally Occurring Substances: Food Items and Constituents, Heterocyclic Aromatic Amines and Mycotoxins. Monograph on the Evaluation of Carcinogenic Risks to Humans.

[8] EFSA (2013). Scientific Opinion on risks for animal and public health related to the presence of nivalenol in food and feed. EFSA J..

[9] Rocha O., Ansari K., Doohan F.M. (2005). Effects of trichothecene mycotoxins on eukaryotic cells: A review. Food Addit. Contam..

[10] Desjardins A.E. (2007). Chapter 1. Trichothecenes. Fusarium Mycotoxins-Chemistry, Genetics, and Biology.

[11] McCormick S., Leonard K.J., Bushnell W.R. (2003). The role of DON in pathogenicity. Fusarium Head Blight of Wheat and Barley.

[12] Rychlik M., Humpf H., Marko D., Dänicke S., Mally A., Berthiller F., Klaffke H., Lorenz N. (2014). Proposal of a comprehensive definition of modified and other forms of mycotoxins including “masked” mycotoxins. Mycotoxin Res..

[13] Zhang H., Wang B. (2015). Fates of deoxynivalenol and deoxynivalenol-3-glucoside during bread and noodle processing. Food Control.

[14] Shin S., Torres-Acosta J.A., Heinen S.J., McCormick S., Lemmens M., Paris M.P., Berthiller F., Adam G., Meuhlbauer G.J. (2012). Transgenic Arabidopsis thaliana expressing a barley UDP-glucosyltransferase exhibit resistance to the mycotoxin deoxynivalenol. J. Exp. Bot..

[15] Schweiger W., Boddu J., Shin S., Poppenberger B., Berthiller F., Lemmens M., Muehlbauer G.J., Adam G. (2010). Validation of a candidate deoxynivalenol-inactivating UDP-glucosyltransferase from barley by heterologous expression in yeast. Mol. Plant Microbe Interact..

[16] Berthiller F., Dall’Asta C., Schuhmacher R., Lemmens M., Adam G., Krska R. (2005). Masked mycotoxins: Determination of a deoxynivalenol glucoside in artificially and naturally contaminated wheat by liquid chromatography-tandem mass spectrometry. J. Agric. Food Chem..

[17] Cirlini M., Generotti S., Dall’Erta A., Lancioni P., Ferrazzano G., Massi A., Galaverna G., Dall’Asta C. (2013). Durum Wheat (*Triticum Durum Desf*.) Lines Show Different Abilities to Form Masked Mycotoxins under Greenhouse Conditions. Toxins.

[18] Kostelanska M., Hajslova J., Zachariasova M., Malachova A., Kalachova K., Poustka J., Fiala J., Scott P.M., Berthiller F., Krska R. (2009). Occurrence of deoxynivalenol and its major conjugate, deoxynivalenol-3-glucoside, in beer and some brewing intermediates. J. Agric. Food Chem..

[19] Poppenberger B., Berthiller F., Lucyshyn D., Sieberer T., Schuhmacher R., Krska R., Kuchler K., Glössl J., Luschnig C., Adam G. (2003). Detoxification of the Fusarium mycotoxin deoxynivalenol by a UDP-glucosyltransferase from *Arabidopsis thaliana*. J. Biol. Chem..

[20] Lemmens M., Scholz U., Berthiller F., Dall’Asta C., Koutnik A., Schuhmacher R., Adam G., Buerstmayr H., Mesterházy A., Krska R. (2005). The ability to detoxify the mycotoxin deoxynivalenol colocalizes with a major quantitative trait locus for Fusarium head blight resistance in wheat. Mol. Plant Microbe Interact..

[21] Li X., Sanghyun S., Heinen S., Dill-Macky R., Berthiller F., Nersesian N., Clemente T., McCormick S., Muehlbauer G.J. (2015). Transgenic wheat expressing a barley UDP-glucosyltransferase detoxifies deoxynivalenol and provides high levels of resistance to *Fusarium graminearum*. Mol. Plant Microbe Interact..

[22] De Nijs M., Van den Top H.J., Portier L., Oegema G., Kramer E., Van Egmond H.P., Hoogenboom L.A.P. (2012). Digestibility and absorption of deoxynivalenol-3-ß-glucoside in in vitro models. World Mycotoxin J..

[23] Berthiller F., Krska R., Domig K.J., Kneifel W., Juge N., Schuhmacher R., Adam G. (2011). Hydrolytic fate of deoxynivalenol-3-glucoside during digestion. Toxicol. Lett..

[24] Pierron A., Mimoun S., Murate L.S., Loiseau N., Lippi Y., Bracarense A.P., Liaubet L., Schatzmayr G., Berthiller F., Moll W.D. (2016). Intestinal toxicity of the masked mycotoxin deoxynivalenol-3-β-d-glucoside. Arch. Toxicol..

[25] Trombete F., Barros A., Vieira M., Saldanha T., Venâncio A., Fraga M. (2016). Simultaneous Determination of Deoxynivalenol, Deoxynivalenol-3-Glucoside and Nivalenol in Wheat Grains by HPLC-PDA with Immunoaffinity Column Cleanup. Food Anal. Methods.

[26] Geng Z., Yang D., Zhou M., Zhang P., Wang D., Liu F., Zhu Y., Zhang M. (2014). Determination of deoxynivalenol-3-glucoside in cereals by hydrophilic interaction chromatography with ultraviolet detection. Food Anal. Methods.

[27] Yoshinari T., Sakuda S., Furihata K., Furusawa H., Ohnishi T., Sugita-Konishi Y., Ishizaki N., Terajima J. (2014). Structural determination of a nivalenol glucoside and development of an analytical method for the simultaneous determination of nivalenol and deoxynivalenol, and their glucosides, in wheat. J. Agric. Food Chem..

[28] Dall’Asta C., Dall’Erta A., Mantovani P., Massi A., Galaverna G. (2012). Occurrence of deoxynivalenol and deoxynivalenol-3-glucoside in durum wheat. World Mycotoxin J..

[29] Dong F., Wang S., Yu M., Sun Y., Xu J., Shi J. (2017). Natural occurrence of deoxynivalenol and deoxynivalenol-3-glucoside in various wheat cultivars grown in Jiangsu province, China. World Mycotoxin J..

[30] Palacios S.A., Erazo J.G., Ciasca B., Lattanzio V.M., Reynoso M.M., Farnochi M.C., Torres A.M. (2017). Occurrence of deoxynivalenol and deoxynivalenol-3-glucoside in durum wheat from Argentina. Food Chem..

[31] Bryła M., Waśkiewicz A., Podolska G., Szymczyk K., Jędrzejczak R., Damaziak K. (2016). Occurrence of 26 Mycotoxins in the Grain of Cereals Cultivated in Poland. Toxins.

[32] Calori-Domingues M.A., Bernardi C.M., Nardin M.S., de Souza G.V., Dos Santos F.G., Stein Mde A., Gloria E.M., Dias C.T., de Camargo A.C. (2016). Co-occurrence and distribution of deoxynivalenol, nivalenol and zearalenone in wheat from Brazil. Food Addit. Contam. Part B Surveill..

[33] De Boevre M., Landschoot S., Audenaert K., Maene P., Di Mavungu D., Eeckhout M., Haesaert G., De Saeger S. (2014). Occurrence and within field variability of Fusarium mycotoxins and their masked forms in maize crops in Belgium. World Mycotoxin J..

[34] Berthiller F., Dall’Asta C., Corradini R., Marchelli R., Sulyok M., Krska R., Adam G., Schuhmaher R. (2009). Occurrence of deoxynivalenol and its 3-β-d-glucoside in wheat and maize. Food Addit. Contam. Part A Chem. Anal. Control Expo. Risk Assess..

[35] Rasmussen P.H., Nielsen K.F., Ghorbani F., Spliid N.H., Nielsen G.C., Jørgensen L.N. (2012). Occurrence of different trichothecenes and deoxynivalenol-3-β-d-glucoside in naturally and artificially contaminated Danish cereal grains and whole maize plants. Mycotoxin Res..

